# Accidental tamoxifen ingestion in a two-year-old child without major immediate symptoms: Case report

**DOI:** 10.1016/j.toxrep.2025.102154

**Published:** 2025-10-30

**Authors:** Alise D.E. de Groot, Willemien F.J. Hof, Hester van Meer, Niels de Vries, Daan J. Touw, Alwin D.R. Huitema, Paola Mian

**Affiliations:** a Department of Pediatrics, Division of Pediatric Gastroenterology and Hepatology, Beatrix Children’s Hospital, University of Groningen, University Medical Center Groningen, Groningen, the Netherlands; b Department of Pharmacy & Pharmacology, Antoni van Leeuwenhoek, Amsterdam, the Netherlands; c Department of Clinical Pharmacy and Pharmacology, University of Groningen, University Medical Center Groningen, Groningen, the Netherlands; d Princess Máxima Center for Pediatric Oncology, Utrecht, the Netherlands; e Department of Clinical Pharmacy, University Medical Center Utrecht, Utrecht University, Utrecht, the Netherlands; f Department of Pediatrics, Beatrix Children’s Hospital, University of Groningen, University Medical Center Groningen, Groningen, the Netherlands

**Keywords:** Anti-cancer drug, Tamoxifen, Child, Poisoning

## Abstract

Tamoxifen is a selective oestrogen receptor modulator indicated for the treatment of breast cancer in adults. The recommended dose is 20 mg orally once daily. We report an accidental tamoxifen ingestion in a 2-year-old female who accessed the tamoxifen from a pill bottle stored in a bag while unsupervised. Activated charcoal and sodium sulphate were administered approximately 2.5 h after ingestion. Plasma concentrations of both tamoxifen and active metabolite endoxifen were determined. The maximum plasma concentration of tamoxifen was 53.8 ng/mL, occurring two hours after ingestion. In contrast, the plasma concentration of endoxifen measured at 22 h after ingestion was 0.771 ng/L, which is considered subtherapeutic in adults. However, given the prolonged half-life of endoxifen, the plasma levels may still be rising at this point. The ingested dose was estimated to be around 31–37 mg, based on the plasma levels of tamoxifen and endoxifen. The actual ingested amount may have been higher due to early oral administration of activated charcoal. No direct serious events occurred during a hospital admission of 31 h. Given the anti-oestrogen properties of tamoxifen and the critical role of oestrogen in pubertal development long-term follow-up is recommended to monitor potential delayed effects.

## Highlights

• A 2-year-old ingested approximately 31–37 mg tamoxifen unsupervised.
• Plasma concentrations are measured to follow the course of the patient.
• No acute adverse events occurred, but long-term monitoring is advised for developmental effects.

## Graphical Abstract

**Figure:**
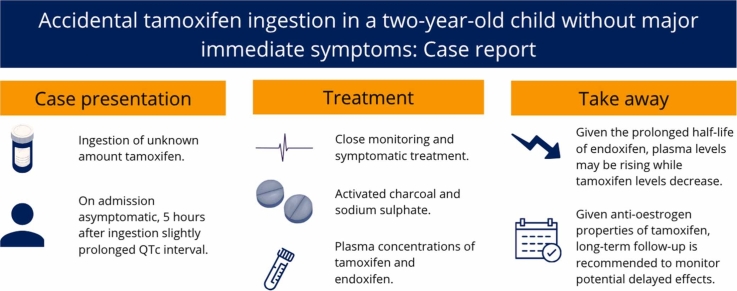

## 1 Introduction

Tamoxifen is an oestrogen receptor modulator primarily used for the treatment and prevention of oestrogen receptor-positive breast cancer in adults [1], [2]. Tamoxifen is widely used in treatment for hormone receptor positive breast cancer and for male gynaecomastia [3]. For both breast cancer and gynaecomastia a dosages of 20 mg per day is recommended [3]. It is also illicitly used by bodybuilders after extended periods of testosterone use [4].

Tamoxifen is metabolized by cytochrome P450 (CYP) 2D6 and CYP3A4 into 4-hydroxy tamoxifen and 4-hydroxy-N-desmethyl tamoxifen (endoxifen). These major active metabolites are 30–100 times more potent and have a longer half-life, 12–14 days compared to tamoxifen itself, which has a half-life of 5–7 days [4]. In adults, tamoxifen administration and presence of metabolites has been associated with adverse effects at dosages ranging from 10 to 30 mg daily, including prolonged QTc intervals, and arrhythmias [5].

Despite extensive literature on the effects of tamoxifen in adults [6], [7], the available literature of tamoxifen toxicity, after accidental intake, in pediatric patients is limited. Literature reports cases of adults patients with high tamoxifen exposure experiencing ocular toxicity, prolonged QTc intervals and ventricular arrhythmias [8], [9], [10]. We report a unique case of an accidental ingestion of tamoxifen in a two-year-old child and the management after this ingestion.

## 2 Case report

A 2-year-old female with a known diagnosis of haemoglobin E thalassaemia was admitted to the emergency department following a suspected accidental ingestion of tamoxifen. Approximately 1.5 h prior to admission, she was left unsupervised and managed to open a bottle of tamoxifen tablets belonging to one of her parents. The exact quantity ingested was unknown, however the parent estimated that she may have ingested between 10 and 20 tablets of 20 mg each, corresponding to a total dose of 200–400 mg. There was no evidence to suggest ingestion of any other substance. The child did not us any other medications during the time of tamoxifen ingestion.

Upon admission, the child was asymptomatic. She had not vomited and remained alert and responsive. During clinical evaluation, she was crying and exhibited resistance to the examination. Her heartrate was 180 per minute, with a respiratory rate of 25 per minute and saturation of 99 %.

**Fig. 1:**
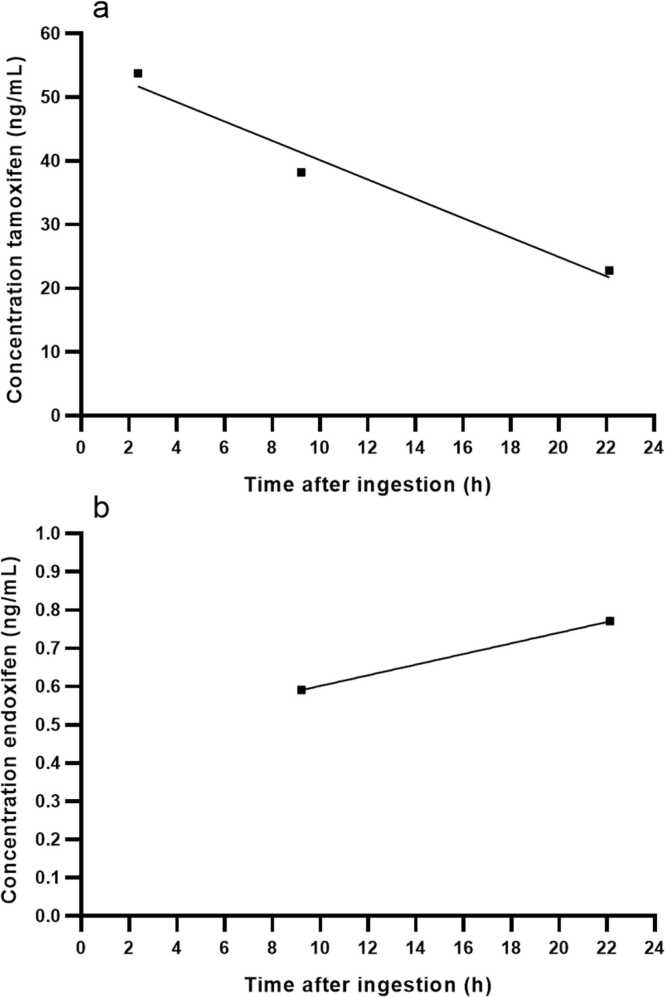
Measured concentrations of tamoxifen and endoxifen in plasma collected in lithium heparin tubes. (a) Concentrations of tamoxifen. (b) Concentrations of endoxifen.

The child received 12.5 g activated charcoal and 6 g sodium sulphate via nasogastric tube 1 h after presenting at the emergency department. Biochemical parameters were within normal ranges at time of admission and remained stable throughout the hospital stay. Electrocardiograms (ECGs) were performed 2 h, 5 h and 23 h after ingestion. No abnormalities were observed in the ECGs 2 h and 25 h after ingestion. However, 5 h after ingestion the QTc interval was slightly prolonged compared to the other ECGs (QTc at 5 h of 462 ms and QTc at 25 h of 380 ms). Blood samples were taken at 2 h, 9 h and 22 h after ingestion for clinical biochemistry. The plasma concentrations of tamoxifen and endoxifen, are measured with HPLC-MS/MS [11]. Plasma concentrations of tamoxifen measured at 2, 9 and 22 h were 53.8 ng/mL, 38.2 ng/mL and 22.8 ng/mL, respectively (Fig. 1**a**). For endoxifen the plasma concentration at 2 h was below the limit of quantification, at 9 and 22 h it was 0.591 ng/mL and 0.771 ng/mL, respectively (Fig. 1**b**). The highest concentration of tamoxifen was measured 2.5 h after ingestion. At 9 h the measured tamoxifen concentration was lower and therefore it was believed that maximum concentration (C_max_) was within the first 9 h after ingestion. The tamoxifen and metabolites concentration measured at 2.5 h of 54.4 ng/mL was used to calculate the probably ingested dose. To calculate the ingested amount of tamoxifen equation 1 is used [12].

(1) $$C_{\max}=\frac{F\;\left(\mathrm{bioavailability}\right)\;\times \;D\;(\mathrm{dose})}{V_{d}\;(\mathrm{volume}\;\mathrm{of}\;\mathrm{distribution})}$$

With a volume of distribution of 50–60 L/kg, the volume of distribution in this patient was between 570 and 690 L [4]. Assuming a bioavailability of 100 %, the ingested quantity of tamoxifen was approximately 31–37 mg, suggesting that the patient had ingested two 20 mg tablets.

The patient was discharged 31 h after admission, as the peak concentrations of tamoxifen had passed without the development of any clinical symptoms. The plasma concentration of endoxifen could still be increasing, because of the prolonged half-life of endoxifen. Three weeks after the tamoxifen poisoning, the patient attended a medical appointment for an unrelated issue. No abnormalities related to the poisoning were reported.

## 3 Discussion

We report a case of a child taking tamoxifen accidentally. Upon admission, the child was asymptomatic. Accidental administration of tamoxifen in a child is rare but may lead to significant adverse effects, including QTc interval prolongation. In this case, the clinical pharmacists recommends 8 h of monitoring for patients with severe symptoms such as significant vomiting or a prolonged QTc interval [13], [14]. The child in this case was monitored in the hospital for 31 h without showing any symptoms, because it was believed to be at increased risk of such symptoms. Firstly, because naïve users may be more susceptible for tamoxifen than regular users. Hence, performing an ECG is always recommended for naïve users [13], [14]. Moreover, a prolonged QTc interval may occur in adults at therapeutic tamoxifen doses, which is 20–40 mg a day for treatment of breast cancer [4]. Secondly, due to decreased CYP2D6 activity in children, tamoxifen is potentially metabolized slower in children than in adults, which may increase the T_max_ [15]. In this case, the observed decline in tamoxifen plasma concentrations over time suggested metabolic activity, and therefore CYP2D6 genotyping was not performed to further investigate the possibility of poor metabolizer status. Madlensky et al. suggest a minimum threshold of 5.97 ng/mL of endoxifen to be considered effective in reducing the risk of recurrence of breast cancer in adult women. The patient in our case had endoxifen concentration of 0.771 ng/mL, the concentration was significantly below the threshold observed in adult women [16]. Nevertheless, close observation of symptoms is needed, given the possibility of endoxifen accumulation.

The ingested dose of tamoxifen was calculated to be approximately 31–37 mg. However, the early administration of activated charcoal probably reduced gastrointestinal absorption and may have led to an underestimation of the actual ingested dose. In adults, steady state plasma concentrations ranging from 0.15 to 0.55 mg/L have been observed following a 40 mg dose of tamoxifen, whereas after single 20 mg dose, the reported C_max_ values for tamoxifen and endoxifen were 0.04 mg/L and 0.015 mg/L, respectively [3], [13]. The maximum plasma concentrations observed in this case (53.5 ng/mL) are comparable to the reported C_max_ values in adults for a single dose of 20 mg. This supports, that it is unlikely that the child ingested the full 20 tablets (equivalent to 400 mg) as initially suspected by the parent.

Additional follow-up may be warranted for children with tamoxifen poisoning depending on the ingested amount. Oestrogen plays a crucial role in the development of secondary sexual characteristics during puberty, such as breast development in girls and the regulation of menstrual cycles [17]. In this case, exposure at an early age to anti-oestrogen drugs may interfere with oestrogen hormonal signalling. Although direct evidence in the pediatric population is limited, a 20-year-old woman who was poisoned with tamoxifen (30 tablets of 20 mg) and diazepam (10 tablets of 5 mg) developed follicular ovarian cysts over the next 4 weeks [16]. These resolved spontaneously within 8 weeks. Four years later, the same patient was admitted because of a tamoxifen (60 tablets of 20 mg) and alcohol (serum level 2.46 mg/mL) poisoning. The patients was 15 weeks pregnant at the time of admission and was alert during admission. Follow-up gynaecological examinations at 5 days and 2 weeks showed no abnormalities, but the patient had a miscarriage almost 5 weeks after the tamoxifen poisoning [18].

Given the critical role of oestrogen in initiating and regulating pubertal development, early tamoxifen exposure in the presented case may carry a risk of long-term reproductive health consequences. Due to limited understanding of tamoxifen’s effect, the potential for endocrine disruption following significant toxic exposure should be considered when assessing long-term outcomes.

In conclusion, we report a case of tamoxifen ingestion in a 2-year-old child, with the exact amount ingested remaining uncertain. Management focused on early absorption prevention and activated charcoal with a laxative, accompanied by close symptomatic monitoring, in line with current treatment recommendations [6]. This strategy, along with monitoring of biochemical parameters proved adequate. The patient remained clinically stable throughout hospitalization. The child was discharged after a 31-h uncomplicated hospital stay. Given the potential for delayed effects, particularly on pubertal and reproductive development, consideration should be given to long-term follow-up.

## CRediT authorship contribution statement

**Alise D.E. de Groot:** Conceptualization, Data curation, Formal analysis, Visualization, Writing – original draft, Writing – review & editing, Validation. **Niels de Vries:** Investigation, Methodology, Writing – review & editing. **Daan J. Touw:** Conceptualization, Investigation, Supervision, Writing – review & editing. **Willemien F.J. Hof:** Conceptualization, Formal analysis, Writing – original draft. **Hester van Meer:** Investigation, Validation, Writing – review & editing. **Alwin D.R. Huitema:** Formal analysis, Methodology, Writing – review & editing. **Paola Mian:** Conceptualization, Data curation, Formal analysis, Investigation, Methodology, Supervision, Writing – original draft, Writing – review & editing.

## Conflict of interest

Alise de Groot, Willemien Hof, Hester van Meer, Niels de Vries, Daan Touw, Alwin Huitema and Paola Mian have no declarations of interest.

## Declaration of Competing Interest

The authors declare that they have no known competing financial interests or personal relationships that could have appeared to influence the work reported in this paper.

## Data Availability

No data was used for the research described in the article.
